# Determination of Dairy Cattle Euthanasia Criteria and Analysis of Barriers to Humane Euthanasia in the United States: Dairy Producer Surveys and Focus Groups

**DOI:** 10.3390/ani10050770

**Published:** 2020-04-29

**Authors:** Brooklyn K. Wagner, Mary Caitlin Cramer, Heather N. Fowler, Hannah L. Varnell, Alia M. Dietsch, Kathryn L. Proudfoot, Jan Shearer, Maria Correa, Monique D. Pairis-Garcia

**Affiliations:** 1 Department of Population Health and Pathobiology, College of Veterinary Medicine, North Carolina State University, Raleigh, NC 27606, USA; 2 Department of Animal Science, College of Agricultural Sciences, Colorado State University, Fort Collins, CO 80523, USA; 3 College of Veterinary Medicine, Iowa State University, Ames, IA 50011, USA; 4 College of Food, Agricultural, and Environmental Sciences, School of Environment and Natural Resources, The Ohio State University, Columbus, OH 43210, USA; 5 Departments of Health Management and Companion Animals, Atlantic Veterinary College, University of Prince Edward Island, Charlottetown, PE C1A 4P3, Canada; 6 Department of Veterinary Diagnostic and Production Animal Medicine, College of Veterinary Medicine, Iowa State University, Ames, IA 50011, USA

**Keywords:** animal welfare, survey, producers, cow, euthanasia

## Abstract

**Simple Summary:**

Making and carrying out euthanasia decisions is a complex, multi-factorial process and effective on-farm euthanasia training tools are needed to increase consistency throughout the dairy industry. This two-part study evaluated the main factors influencing euthanasia practices on dairy farms across the United States using dairy producer surveys (Part I) and focus groups (Part II). Survey results suggest that farm owners are most commonly responsible for making and carrying out euthanasia decisions on-farm and most dairy farmers treat and monitor compromised cattle for a variety of health conditions. Non-ambulatory cattle represent a severely compromised subpopulation of animals; however, they were selected to never be euthanized by 6.3% and 11.7% of survey respondents, respectively. Focus group discussions identified three main themes and 15 subthemes including animal, human and farm operation factors. Animal welfare and health status were frequently discussed and participants readily recognized the multi-factorial nature of on-farm euthanasia. Dairy producers are nuanced in their thinking about euthanasia decision making. However, the high variability in euthanasia timelines represents a significant animal welfare challenge. Training programs that focus on implementing specific standards for euthanasia is a critical next step and more research is needed to understand the factors influencing producer psychological perspectives regarding euthanasia decision making.

**Abstract:**

There are currently no clear guidelines in the US and some other countries regarding euthanasia decision making timelines for dairy cattle that become injured or ill to the extent that recovery is unlikely or impossible. Our study aimed to identify decision making criteria and the most common factors considered when making and carrying out euthanasia decisions. Dairy producers were recruited to participate in a mailed survey (Part I, 307 completed surveys were returned) or in one of three focus groups (Part II, 8–10 producers/group, *n* = 24). Part I (survey): Farm owners were most commonly responsible for on-farm euthanasia and most respondents would treat and monitor compromised cattle for a majority of 15 health conditions. Responses were highly variable; for example, 6.3% and 11.7% of respondents would never euthanize a non-ambulatory cow or calf, respectively. Part II (focus groups): Three main themes (animal, human, and farm operation) were identified from discussion which focused primarily on animal welfare (16% of the discussion) and human psychology (16%). Participants expressed a desire to eliminate animal suffering by euthanizing, alongside a wide range of emotional states. Development of specific standards for euthanasia is a critical next step and more research is needed to understand the human emotions surrounding euthanasia decision making.

## 1. Introduction

Euthanasia is a necessary act for any operation raising animals and performing euthanasia in a timely manner is critical to reducing poor welfare outcomes [1,2]. The act of performing euthanasia is a multi-step process requiring those working with cattle to have the ability to identify compromised animals through observation and the technical skills and willingness to perform humane euthanasia. Dairy producers in the US have access to euthanasia guidelines through associations such as the National Milk Producer’s Federation [3] and the American Association of Bovine Practitioners [4]. However, these guidelines only provide basic information on euthanasia techniques and include limited information regarding euthanasia decision making. Although these guidelines and resources are available, implementing timely euthanasia may be problematic if information is difficult to follow or incorrectly interpreted.

In addition, making and carrying out euthanasia decisions is a complex process that can elicit emotional reactions from animal caretakers and producers. Although we have terminology to describe the emotional strain experienced by individuals tasked with euthanizing animals, such as “caring-killing paradox” [5,6] and “compassion fatigue” [7], little work has been done to date to identify the specific emotional factors at play within the dairy industry. Identifying such factors is vital to addressing the needs of producers and caretakers and facilitating confidence to increase overall willingness and ability to perform euthanasia when cattle become compromised. 

Compromised cattle that fail to receive appropriate assessment and timely euthanasia represent a critical population of animals on-farm [1,2]. Cases of timely euthanasia and prolonged suffering in dairy cattle not only negatively impact the individual animal’s welfare but also result in reduced consumer trust and directly jeopardize the viability of all animal agriculture industries. Moving forward, providing producers with science-based guidelines and accessible training methods will be critical to ensuring timely and humane on-farm euthanasia. However, identifying and understanding the many factors considered by producers throughout the entire euthanasia process is critical to developing any future guidelines or training programs. Therefore, as a two-part series, our study aimed to identify decision making criteria for on-farm euthanasia and the most common factors considered when making and carrying out euthanasia decisions on-farm utilizing survey and focus groups. 

## 2. Materials and Methods

All research was reviewed and approved by North Carolina State University IRB Committee for Human Subjects Research. 

### 2.1. Part I: Survey 

#### 2.1.1. Development and Participant Recruitment

A survey was developed utilizing methods described by Habing et al. [8] and adapted from a previously published survey specific to swine euthanasia developed by Mullins et al. [4]. Content validity of the survey was independently reviewed by five of the co-authors, including experts in euthanasia, cattle health and survey methodology. Health conditions listed within the survey were based on and adapted from a 2014 USDA report identifying the most common health problems reported by US dairy producers as well as from co-authors [9]. 

The National Dairy Farm Program solicited interest from co-ops across the US to participate in the study. Surveys were either mailed to co-ops for distribution to producers or mailed directly to producers. A total of 3664 surveys were mailed to producers across the United States. 

#### 2.1.2. The Survey

The survey (please see Appendix A Table A1 for a full list of survey questions) collected demographic information including age, gender, years of experience working with dairy cattle, education level, cattle inventory information and respondent’s role on the dairy facility. In addition, respondents were asked to provide information regarding the role of the individual primarily responsible for making on-farm euthanasia decisions and performing euthanasia. If “other” was indicated, they were asked to specify with a text response. 

Respondents were also asked to make decisions regarding the management of 15 designated conditions over three production stages (13 conditions specific to adult cows and 9 conditions specific to weaned heifers and pre-weaned calves; some conditions overlapped between age groups). Conditions were randomized within each list to control for order bias or concatenated questions. Respondents selected options based on how they would manage a given condition. The scoring system presented four discrete choices organized in a unipolar matrix design and adapted from Mullins et al. [10]. Choices included euthanize immediately (EI), treat and monitor for signs of improvement (T/M), cull/sell for beef (C/S) and not applicable (N/A). If respondents selected the T/M choice, they were provided with additional space to identify situations in which they would never euthanize due to this condition alone. 

Completed and returned surveys were coded and entered manually into an electronic database by one researcher to support coding consistency and control for inter-individual bias. The likelihood of response bias was minimal given that no identifying information was collected, and respondents remained anonymous. Respondents were able to complete as much or as little of the survey as they wanted, in accordance with IRB protocol. 

#### 2.1.3. Data Analysis 

Data quality and assurance was done to reduce data entry and/or coding errors during and after data transfer into a spreadsheet. Non-response for any question was set as a missing value. Free text responses provided after selection of “other” were coded based on veterinarian involvement (i.e., 1 = euthanasia decisions made by multiple individuals with a veterinarian, or 2 = euthanasia decisions made by multiple individuals without a veterinarian), and were represented as nominal variables for further analysis. Numerical data were checked for possible data gaps and need for categorization was considered. Descriptive statistics were obtained for all survey variables, including frequency for nominal or ordinal data, and mean, standard deviation, median and range for continuous variables. T-tests were used to assess demographic data for differences in relation to respondent role in on-farm euthanasia. The data analysis for this paper was generated using SAS software (^©^ 2020 SAS Institute Inc., version 9.4, Cary, NC, USA).

### 2.2. Part II: Focus Group 

#### 2.2.1. Development and Participant Recruitment

Dairy cattle producers were targeted via three dairy producer conferences held in the US. With support from conference organizers, a recruitment email was sent to registered dairy producers. In order to participate in a focus group, participants were required to have experience as a dairy farm caretaker and to be familiar with the usual euthanasia practices and related training provided on a dairy farm. Interested individuals contacted the research team and a time for focus groups was arranged during each of the three conferences. Focus group participation was optional and voluntary. 

#### 2.2.2. Focus Group Format

A total of three focus groups were conducted by one trained researcher to eliminate any bias from inter-individual differences that can affect the direction and/or outcome of focus groups (e.g., body language, expansion questions asked, pace, etc.). This trained researcher was a postdoctoral research fellow with extensive experience in the dairy industry. Consent forms were obtained for each individual participant and were signed prior to participation in the focus group discussion. In addition, participants were asked to provide demographic information including gender and years of experience in the dairy industry. An incentive ($100 Visa gift card) was mailed to focus group participants within three months of participation.

Discussion was prompted by eight main questions and follow-up questions were asked when deemed necessary by the researcher leading the focus group. For consistency, all focus groups were conducted for a maximum of one hour. The standard questions posed to focus groups participants are provided in Table 1. Focus group discussions were audio recorded and transcribed verbatim by trained individuals. 

#### 2.2.3. Discussion Analysis

An inductive coding strategy with a basis in grounded theory was used to identify focus group discussion main themes and subthemes. This approach was used to prevent *pre-hoc* guidance of discussion interpretation and influence of inherent researcher bias. Transcribed focus group discussions were systematically analyzed by two independent coders who discussed all coding discrepancies until code agreement was reached. Coder 1 is a public health veterinarian and PhD level researcher with experience and expertise in mixed methods approaches with an emphasis on qualitative data analysis. Coder 2 is a postdoctoral research scholar in animal welfare with a PhD and expertise in cattle-focused research.

The proportion of the total discussion dedicated to each subtheme was quantified by identifying the number of times each subtheme was mentioned and dividing this number by the total number of subtheme mentions (e.g., number of times “herd impact” was coded/number of times subthemes were coded: 5/242 = 2.1%). Results are presented as “percent of discussion.” 

## 3. Results

### 3.1. Part I: Demographics

A total of 3664 surveys were sent out and 307 surveys were completed and returned, yielding a response rate of 8.4%. A majority (82.9%) of respondents identified as males, 15.7% as females and 1.3% preferred not to answer. Survey respondent’s median age was 51 years (19 to 84 years). The median number of years of experience with dairy cattle was 40 with a wide range of experience between 1 to 72 years. Levels of experience between males and females were consistent. Most respondents indicated that their highest level of education was high school diploma/GED (35.9%). In addition, 19.2% of respondents either chose not to answer or provided a response that was not available in the multiple-choice options (entered as missing for analysis). Most respondents were farm owners (90.9%), as opposed to farm managers (7.52%), or animal caretakers/employees (<1%). 

**Figure 1:**
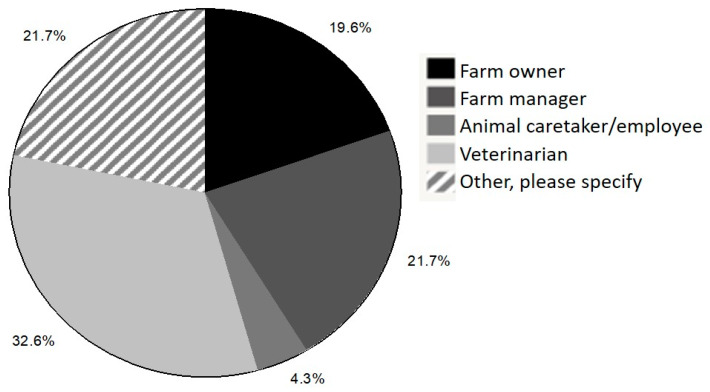
Responses of survey respondents who indicated that they were not the primary party responsible for making euthanasia decisions and were asked to specify the role of the individual who makes most of the euthanasia decisions.

**Figure 2:**
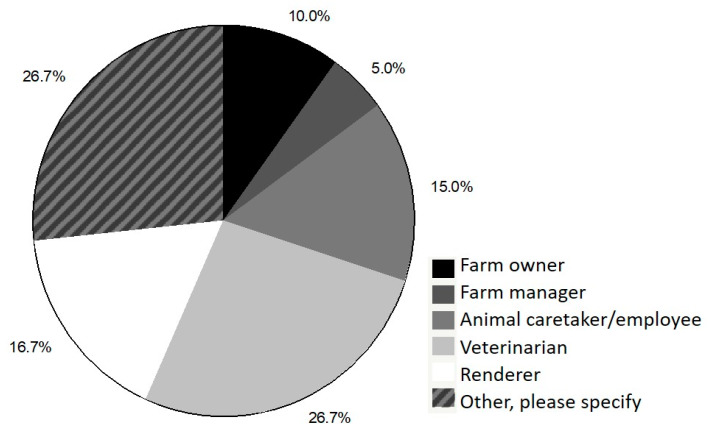
Responses of survey respondents who indicated that they were not the primary party responsible for performing euthanasia and were asked to specify the role of the individual who performs euthanasia.

In the past 12 months, 66.7% of respondents indicated that dairy cattle had been euthanized on the facility where they currently worked and 92.6% of euthanized cattle were adult cows. When asked if respondents were the primary individual responsible for making euthanasia decisions and/or performing euthanasia, 84.8% indicated that they were responsible for making euthanasia decisions and 59.2% were responsible for performing euthanasia. Respondents who were not the primary individual responsible for making and/or carrying out euthanasia decisions were asked to select who was responsible for these actions (Figure 1 and Figure 2). 

If respondents selected “other”, space was provided for them to add additional information to specify who was responsible. Overall, 45.6% and 34.7% of respondents who selected “other” indicated that veterinarians were directly consulted when making and carrying out individual euthanasia decisions on-farm, respectively.

Respondents who indicated that they were the individual primarily responsible for performing euthanasia were most likely to be male (*p* < 0.001) with similar years of experience (*p* = 0.12) and formal education level (*p* = 0.12) as respondents who indicated that someone else was primarily responsible for performing euthanasia. In addition, 96% of respondents who indicated that they were primarily responsible for performing on-farm euthanasia also indicated that they were the individual primarily responsible for making euthanasia decisions.

### 3.2. Part I: Decision Making about Health Conditions

**Figure 3:**
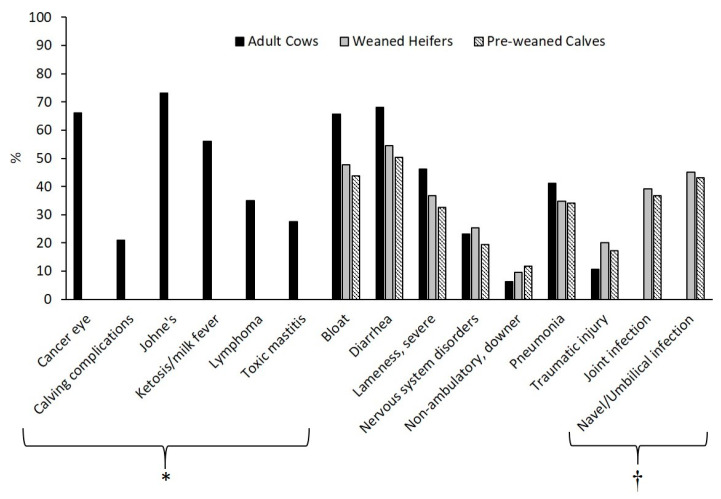
The percentage of respondents who, after choosing the T/M condition management option, indicated that they would never euthanize due to these conditions alone for adult cows, weaned heifers and pre-weaned calves. * These conditions (*n* = 6) were only included for adult cows. † These conditions (*n* = 2) were only included for weaned heifers and pre-weaned calves.

Questions and respondent responses regarding the management of specific conditions for each production stage are provided in Table 2. Regardless of production stage, most respondents indicated that they would treat and monitor for all health conditions considered in the present study with the exception of four conditions. In adult cattle, most respondents selected to cull/sell for beef for cancer eye, Johne’s Disease and lymphoma (35.6%, 68.5% and 51.3%, respectively) and euthanize for traumatic injury for all production stages (43.3%, 45.4%, 43.8% for adult cows, weaned heifers and pre-weaned calves). The percentage of respondents who, after choosing T/M, indicated that they would never euthanize due to these conditions alone are provided in Figure 3.

### 3.3. Part II: Focus Group Participation and Outcomes

Twenty-four dairy producers participated in one of three focus groups (8–10 producers/group). Three main themes (animal, human, and farm operation) and 16 subthemes were identified and are presented in Table 3. Most of the discussion focused on animal factors (46% of the conversation), followed by human factors (32.6%) and farm operation factors (21.5%). 

Briefly, animal factors were comprised of any discussion that focused on the individual animal or the animals on the farm. For example, in response to Q4 (*When do you know it is the right time to euthanize an animal?*) one participant stated that, “If she has a broken leg, the welfare of that animal is compromised to where she’s not going to improve, then that’s when we’re going to decide to euthanize.” This quote, with mention of a broken leg (Animal Factor Subtheme 2: *Health status/condition/disease*), animal welfare (Animal Factor Subtheme 1: *Animal welfare/well-being*) and the animal’s likelihood of improvement (Animal Factor Subtheme 3: *Improvement*), highlights three of the six animal factor subthemes. 

Human factors were discussed frequently and comprised of subthemes ranging from individual feelings about euthanasia to morality, food safety and concerns about public perception. One participant even used the term “human factor” when discussing the drawbacks of euthanizing animals on-farm. On a whole, producers collectively expressed the substantial influence that the human component has in the euthanasia process.

Lastly, farm operation factors were named as such to encompass any discussion pertaining to the logistics of on-farm euthanasia. For example, carcass disposal and cost (e.g., disposal charges, negative return on investment, etc.) considerations were frequently cited as on-going challenges to euthanizing animals on-farm. However, use of written, on-farm protocols was prevalent and often discussed as a critical resource to ensure that, “The people we have assigned to [euthanize] are doing it in the proper, most humane way possible.” Overall, farm operation factors, though containing many subthemes, represented the smallest proportion of focus group discussions. 

## 4. Discussion

The objective of the present study was to use survey and focus group methodologies to identify both the current decision-making criteria being used by dairy producers and the most common factors considered when making and carrying out euthanasia decisions on-farm. When cattle become severely ill or injured on dairy farms, performing euthanasia is a critical tool used to reduce the incidence of poor welfare outcomes [2,11]. The complex, multi-step euthanasia process requires skilled individuals to identify compromised animals, administer appropriate treatment, and if necessary, humanely euthanize if likelihood of recovery is poor [12,13]. However, information is deficient for providing dairy producers with guidelines and training resources specific to timely and humane euthanasia. This gap in resources may be contributing to the circumstances identified in the present study including (1) a disproportionate reliance on farm owners and external input when making and carrying out euthanasia decisions, (2) compromised emotional well-being of animal caretakers and (3) inconsistencies in the management of health conditions. 

### 4.1. Reliance upon Farm Owners and External Guidance

The present study identified farm owners as the individuals most commonly responsible for the on-farm euthanasia process and it remains unclear if this role is taken on by choice or out of necessity. Milk production is labor intensive and requires skilled individuals to support animal husbandry and milk processing needs [14]. However, skilled labor is expensive and often unavailable or at a high turnover rate in rural areas of the country [15]. Therefore, farm owners and producers often take on and prioritize key management areas for the farm such as animal care and management [16], under which euthanasia would fall.

Given that 45.6% and 34.7% of respondents indicated that veterinarians were directly consulted when making and carrying out individual euthanasia decisions, respectively, some producers do not feel fully equipped to conduct the euthanasia process without accessing external resources. Relying on the veterinarian to weigh-in on all euthanasia decisions may result in poor welfare outcomes due to the veterinarian’s limited availability to assist in a timely manner when a euthanasia case presents. However, from a welfare perspective, this consultation may be important given that veterinarians are the group most likely to have received formal training on euthanasia [17].

Veterinarians are not financially impacted by animal loss thus minimizing or preventing inappropriate euthanasia decisions that are economically driven by the producer [18]. Focus group participants readily acknowledged their desire to, “Get a little bit of the investment back out of that cow,” and identified that cull prices were often considered when making euthanasia decisions. Producers make euthanasia decisions not only for the welfare of the cow but must also consider the cost associated with losing an animal in an economic state where average profit and individual cow value is low [19]. However, most participating dairy producers also expressed a willingness to lose money as opposed to shipping compromised cattle or allowing cattle to suffer unnecessarily. The desire to be a ‘responsible producer’ was frequently mentioned regarding why a producer would choose to hold what is best for the animal in higher regard than what is economically beneficial. 

Providing science-based standards, clear and comprehensive guidelines, and accessible training tools supports farm owners in their commitment to be “responsible producers” by allowing them to feel confident in their ability to objectively consider all factors that come into play when making and carrying out on-farm euthanasia.

### 4.2. Human Psychology and Emotions

Focus group participants in the present study expressed a wide range of emotional states regarding the topic of on-farm euthanasia including moral obligation, attachment to the animal and profound feelings of failure when cattle required euthanasia. Many participants made statements referencing morality (e.g., “It’s the right thing to do”) and/or the presence of an ethical responsibility to the animal.

A general sense of failure was evident among participating dairy producers when discussing euthanizing compromised cattle. One individual stated “[I]t gives me a sense of failure because we failed on that cow somehow or another.” The feeling of failure expressed here may be a result of well-defined concepts such as the “caring-killing paradox” [3,4], “moral stress” [5] and “compassion fatigue” [6]. Briefly, these concepts outline the mental and emotional strain experienced by individuals tasked with euthanizing animals. Some participants even referenced spirituality coming into play when faced with on-farm euthanasia, stating “[W]e’ve never euthanized an animal on our farm without having that spiritual conversation…”.

While it is heartening to confirm that dairy producers care deeply about their animals, the considerable emotion experienced by individuals involved in the euthanasia process is an important factor that must be considered when tasking individuals with such a duty on-farm. Moreover, awareness of the human factor, supported by the present study, can aid in the development of guidelines and training materials. This proactive approach may reduce incidences of compromised mental health in caretakers, ultimately minimizing labor turnover rates and supporting farm profitability.

### 4.3. Inconsistent Health Condition Management and Euthanasia Timelines

The present study identified that euthanasia is not a primary tool considered when an animal presents with clinical signs of disease. Dairy cattle health presents on a continuum and can be challenging to assess given that clinical signs and physiological changes associated with disease can vary dramatically. Clinical presence of a disease depends significantly on the severity of the infection/strain, immune status of the animal and disease chronicity [20]. Acute disease states, such as those experienced with diarrhea, ketosis and milk fever, will often present with obvious clinical signs such as anorexia, severe lethargy and fever [21]. However, cattle experiencing a chronic disease state, such as cancer eye, lymphoma and lameness, often exhibit vague behavioral and physiological responses, with evidence of gradual loss of body condition or productivity over time. Therefore, it may be difficult for farm owners to identify, appropriately select and treat cattle effectively with limited knowledge or background in veterinary medicine or animal health [22]. These challenges threaten industry consistency and can result in greater incidences of poor welfare outcomes.

Additionally, the value of treatment for many of the health conditions investigated in the survey remains unclear. Much work has been conducted in determining effective treatment outcomes for severe conditions such as lameness, cancer eye, non-ambulatory and bloat [22] with most studies revealing that these conditions are considered refractory to treatment unless identified and treated as the earliest sign of disease [9,23,24,25]. Yet, 28.4% of respondents would elect to treat and monitor adult cows with cancer eye, 59.7–68.3% would treat and monitor cattle of all production stages demonstrating severe lameness and 46.3–54.9% for non-ambulatory cattle. Although treatment may not be the best option for animals experiencing these health conditions, results of the present study indicate that this is the option that farm owners are most comfortable making, thus increasing treatment prevalence across all stages of production unnecessarily. Mullins et al. [10] report a similar phenomenon in swine producers, highlighting their hope that additional treatment, despite treatment duration or success, will help the pig to improve. Given the influence of human psychology and emotions identified by focus group discussion analysis in the present study, it is possible that this proclivity to treat animals may be grounded in the farm owners equating euthanasia with a failure to provide adequate care [26].

Ambiguity in treatment timelines and euthanasia decisions was also prevalent in focus group discussions. Specifically, management of non-ambulatory cattle represented a large portion of the discussion. Non-ambulatory, or “downer” cattle, characterized as sternal recumbency for ≥12 h [27], represent a significant animal welfare issue for the dairy industry. In 2013 alone, an estimated 2.6% of all US dairy cows became non-ambulatory [28]. Given that only one-third of down cows ever recover (only 8% if down for ≥24 h) [11], providing prolonged treatment is not appropriate practice and down cattle should be euthanized in a timely manner.

Although down cattle were identified frequently as animals requiring euthanasia, timelines discussed amongst focus group participants were vague and inconsistent. For example, one participant stated, “We have between 28 and 48 h…” in reference to down cows, while others stated, “…[A]fter 48 h she really has to show improvement on a day-to-day basis…”, or “…[W]henever we have a down cow we evaluate her at the next manager’s meeting.” The inappropriate treatment and delayed decision to euthanize non-ambulatory animals is not only a welfare issue but can also have serious economic implications to producers given the cost of treatment and labor required to manage down cattle appropriately [22].

Inappropriate or untimely treatment becomes an even greater concern when severely compromised cattle remain on the farm and can become potential candidates for transport to cull plants or die without assistance [18]. In addition to respondents that would treat non-ambulatory cattle, 3.4%–6.5% would cull/sell non-ambulatory cattle for beef and, of those cattle in which treatment was not successful, 6.3%–11.7% would never euthanize a down cow or calf.

These results not only directly contradict fitness for transport guidelines (i.e., non-ambulatory or severely lame animals should not be transported; US: Farmers Assuring Responsible Management and the American Association of Bovine Practitioners) and regulations (Europe: Directive 91/628/EEC, “Animals that are ill or injured shall not be considered fit for transport”) set out by the industry, but identify a significantly compromised subpopulation of animals which require and fail to receive immediate euthanasia. Calculations reported by Walker et al. [22] identified severe lameness and non-ambulatory as two severe conditions which warrant euthanasia. Based on a lactating cow population of 9,399,000, over 250,000 dairy cattle in the US with severe lameness or non-ambulatory status were marketed when they should have been euthanized. Acknowledging these significant welfare concerns and identifying the risk that these animals pose to the US dairy industry, development of clear guidelines for conditions that warrant immediate euthanasia are needed.

## 5. Conclusions

In conclusion, producers understand the value of utilizing euthanasia to eliminate pain and suffering and place emphasis on the welfare of the animal over economic considerations. However, given the significant inconsistencies regarding timely and appropriate euthanasia timelines, occurrences of poor animal welfare outcomes for compromised cattle are substantial. Moving forward, training tools need to be developed that provide clear standards regarding treatment and euthanasia timelines that allow producers to confidently and independently practice on-farm euthanasia when necessary. More research is needed to address the psychological and emotional impacts of euthanasia on dairy producers and caretakers.

## Figures and Tables

**Table 1:** Questions utilized in a focus group discussion on timely euthanasia on dairy farms in the US.

Q#	Question
Q1	What comes to mind when you think about euthanizing animals on-farm?
Q2	What, if any, are the benefits of euthanizing animals on-farm?
Q3	What, if any, are the drawbacks to euthanizing animals on-farm?
Q4	When do you know it is the right time to euthanize an animal?
Q5	When do you know it is NOT the right time to euthanize an animal?
Q6	What are the main reasons why you would delay euthanasia?
Q7	What are the main reasons why you would NOT perform euthanasia?
Q8	What other factors might you consider when making the decision to euthanize animals on-farm?

**Table 2:** Response percentages of surveyed dairy cattle producers (*n* = 270) when asked to select how they would manage the following conditions.

Condition	Euthanize Immediately (%)	Treat and Monitor for Signs of Improvement (%)	Cull/Sell for Beef (%)	N/A (%)
**Adult Cow**				
Bloat	0.36	81.1	4.63	13.9
Cancer eye, severe	3.64	28.4	35.6	32.4
Calving complications ^1^	2.69	86.5	7.31	3.46
Diarrhea ^2^	-	79.6	12.2	8.15
Johne’s Disease	0.73	10.3	68.5	20.5
Ketosis / Milk fever	-	97.2	1.40	1.40
Lameness, severe ^3^	1.24	61.6	35.1	2.07
Lymphoma ^4^	8.12	8.49	51.3	32.1
Nervous system disorders ^5^	21.1	27.6	28.0	23.3
Non-ambulatory / Downer	36.4	54.9	6.55	2.18
Pneumonia	-	88.9	7.89	3.23
Toxic mastitis	4.75	57.3	35.0	2.92
Traumatic injury	43.3	28.9	18.6	9.13
**Weaned Heifers**				
Bloat	0.73	83.9	3.65	11.7
Diarrhea	-	87.3	4.36	8.36
Joint infection	2.61	67.2	19.4	10.8
Lameness, severe	3.45	59.7	28.3	8.53
Navel / Umbilical infection	0.362	87.0	3.26	9.42
Nervous system disorder	23.4	32.3	20.4	23.8
Non-ambulatory / Downer	40.9	48.3	4.83	5.95
Pneumonia	0.38	92.5	4.12	3.00
Traumatic injury	45.4	32.7	12.7	9.23
**Pre-weaned Calves**				
Bloat	1.10	82.4	1.10	15.4
Diarrhea	-	93.8	1.82	4.36
Joint infection	2.93	76.9	7.33	12.8
Lameness, severe	2.99	68.3	13.1	15.7
Navel / Umbilical infection	0.73	89.0	1.83	8.43
Nervous system disorder	29.3	34.6	11.4	24.7
Non-ambulatory / Downer	39.6	46.3	3.36	10.8
Pneumonia	0.73	93.4	1.83	4.03
Traumatic injury	43.8	37.7	6.53	11.9

^1^ Paralysis, dystocia, prolapsed uterus, C-section. ^2^ Severe, with dehydration. ^3^ Severe; score of 3 on 3-point scale; score 4 on 4-point scale; score 5 on 5-point scale. ^4^ Bovine leukosis. ^5^ Circling or incoordination; convulsions; involuntary eye movement; head tilt.

**Table 3:** Main themes and subthemes discussed by focus group participants, the proportion of time dedicated to each theme and a brief, direct quote that highlights each theme.

Themes	% of Discussion	Direct Quotes
Animal Factors		
Animal welfare/well-being	16	“You are removing the pain and suffering from that animal, so they don’t have to endure whatever it is that you’re euthanizing them for...”
Health status/condition/disease	15	“[L]isten to what the animal is telling you...their state of physical health.”
Improvement	11	“[C]ompromised to where she’s not going to improve, that’s when we’re going to decide to euthanize.”
Herd impact	2.1	“By removing that animal from the group, you are adding back to the other animals.”
Transport survivability	1.2	“We’re very conscious of not sending animals to slaughter that have a condition that would not allow them to survive the trip…”
Productivity potential	1.2	“[I]f this animal is not going to perform to possibly your standards or the average in the herd.”
Human Factors		
Emotions/psychology	16	“[I] know it’s the right thing, but it is a tough thing.”
Human safety/food safety	7.4	“Safety for everybody involved.”
Education/training	5.4	“[M]ake sure that we have our employees trained to do that correctly as well as properly euthanize that animal without causing her anymore suffering.”
Public perception	3.7	“[V]isually to somebody that doesn’t understand what’s going on, it’s a PR issue and I would say that is a drawback.”
Farm Operation Factors		
Financial/economical	7.4	“[I]f cull prices are up you’re obviously going to do more to try and get that animal into a state where it can be sold rather than be euthanized.”
Protocols/procedures/ guidelines	6.2	“[B]eing able to do it ourselves on the farm, following our strict protocols that we’ve developed with our veterinarian…”
Carcass disposal	2.1	“[D]isposal of the animal is always a problem and timely disposal too.”
Equipment	2.1	“[W]hether you use a gun or a deadbolt, those tools, if not used correctly, can create a potentially unsafe environment…”
Veterinarian recommendation	2.1	“If you think you’ve got a disease present and you need to delay so that the vet has time to test to figure out what disease you’re dealing with…”
Time/labor/space	1.7	“[I]t’s also the time and effort herdsman and other people…that we work with have put time and effort into an animal if it’s a sick animal…”

## Appendix A

**Table A1:** Survey questions used to attain respondent and farm demographic information, including the response options provided for each question.

Q#	Question Text	Response Options
2.1	What is your age?	*Text box provided*
2.2	What is your gender?	Male Female Other Prefer not to answer
2.3	Approximately how long have you worked with dairy cattle?	*Text box provided*
2.4	What is your highest level of education?	Some high school High school diploma/GED Associates degree/certificate Bachelor’s degree Graduate degree (e.g., MS, MBA, PhD)/ professional degree (e.g., DVM)
2.5	Approximately how many adult cows (lactating and dry) are on the facility where you currently work?	*Text box provided*
2.6	Approximately how many heifers and calves are on the facility where you currently work?	*Text box provided*
2.7	With which of the following cattle groups do you currently work? *(select all that apply)*	Pre-weaned calves Weaned heifers Mature cows Bulls
2.8	Which of the following best describes your role on the dairy facility where you currently work?	Farm owner Farm manager Animal caretaker/employee Veterinarian Other, please specify: *Text box provided*
2.9	In the past 12 months, have any dairy cattle been euthanized on the facility where you currently work?	Yes No
	*If yes* → Q2.10: Which groups of dairy cattle have you euthanized? *(select all that apply)*	Dairy bulls or dairy yearling bulls Dairy steers/beef Pre-weaned calves (calves still on milk) Weaned heifers Adult cows
2.11	Who performs most euthanasia on the facility where you currently work?	I do Someone else (see Figure 1)
	*If someone else* → Q2.12: If someone else performs most euthanasia, what is this person’s role?	Farm owner Farm manager Animal caretaker employee Veterinarian Renderer, how frequently does the renderer come to this facility?: *Text box provided* Other, please specify: *Text box provided*
2.14	Who makes most of the decisions on the facility to euthanize an animal?	I do Someone else (see Figure 2)
	*If someone else* → Q2.15: If someone else makes most of the decisions to euthanize an animal, what is this person’s role?	Farm owner Farm manager Animal caretaker /employee Veterinarian Other, please specify: *Text box provided*
2.16	Do you have a written protocol for dairy cattle euthanasia on the facility where you currently work?	Yes No
	*If yes* → Q2.17: Was the written euthanasia protocol developed in consultation with the farm veterinarian?	Yes
		No
2.18	In the past 12 months, how often was the farm veterinarian consulted before euthanizing dairy cattle (lactating dairy cows, dairy heifers, dairy calves) on the facility where you currently work?	Always/every case
		Often/most cases
		Sometimes/a few cases
		Never/no cases
